# Chronic HIV-1 Infection Alters the Cellular Distribution of FcγRIIIa and the Functional Consequence of the FcγRIIIa-F158V Variant

**DOI:** 10.3389/fimmu.2019.00735

**Published:** 2019-04-10

**Authors:** Ntando G. Phaahla, Ria Lassaunière, Bianca Da Costa Dias, Ziyaad Waja, Neil A. Martinson, Caroline T. Tiemessen

**Affiliations:** ^1^Centre for HIV and STIs, National Institute for Communicable Diseases, Johannesburg, South Africa; ^2^Faculty of Health Sciences, University of the Witwatersrand, Johannesburg, South Africa; ^3^Department of Virus and Microbiological Special Diagnostics, Statens Serum Institut, Copenhagen, Denmark; ^4^Perinatal HIV Research Unit, University of the Witwatersrand, Johannesburg, South Africa; ^5^MRC Soweto Matlosana Centre for HIV/AIDS and TB Research, Johannesburg, South Africa

**Keywords:** NK cells, CD8 T cells, antibody-dependent cellular cytotoxicity, Fc gamma receptor, polymorphism, HIV, infection

## Abstract

Chronic HIV-infection modulates the expression of Fc gamma receptors (FcγRs) on immune cells and their antibody-dependent effector function capability. Given the increasingly recognized importance of antibody-dependent cellular cytotoxicity (ADCC) in HIV-specific immunity, we investigated the cellular distribution of FcγRIIIa on cytotoxic lymphocytes—natural killer cells and CD8^+^ T cells—and the effect of the FcγRIIIa-F158V variant on ADCC capacity in HIV-infected individuals (*n* = 23) and healthy controls (*n* = 23). Study participants were matched for F158V genotypes, carried two copies of the *FCGR3A* gene and were negative for FcγRIIb expression on NK cells. The distribution of CD56^dim^FcγRIIIa^bright^ and CD56^neg^FcγRIIIa^bright^ NK cell subsets, but not FcγRIIIa surface expression, differed significantly between HIV-1 negative and HIV-1 positive donors. NK cell-mediated ADCC responses negatively correlated with the proportion of the immunoregulatory CD56^bright^FcγRIIIa^dim/neg^ cells and were lower in the HIV-1 positive group. Intriguingly, the FcγRIIIa-F158V variant differentially affected the NK-mediated ADCC responses for HIV-1 negative and HIV-1 positive donors. Healthy donors bearing at least one 158V allele had higher ADCC responses compared to those homozygous for the 158F allele (48.1 vs. 34.1%), whereas the opposite was observed for the HIV-infected group (26.4 vs. 34.6%), although not statistically significantly different. Furthermore, FcγRIIIa^+^CD8^bright^ and FcγRIIIa^+^CD8^dim^ T cell subsets were observed in both HIV-1 negative and HIV-1 positive donors, with median proportions that were significantly higher in HIV-1 positive donors compared to healthy controls (15.7 vs. 8.3%; *P* = 0.016 and 18.2 vs. 14.1%; *P* = 0.038, respectively). Using an HIV-1-specific GranToxiLux assay, we demonstrate that CD8^+^ T cells mediate ADCC through the delivery of granzyme B, which was overall lower compared to that of autologous NK cells. In conclusion, our findings demonstrate that in the presence of an HIV-1 infection, the cellular distribution of FcγRIIIa is altered and that the functional consequence of FcγRIIIa variant is affected. Importantly, it underscores the need to characterize FcγR expression, cellular distribution and functional consequences of FcγR genetic variants within a specific environment or disease state.

## Introduction

Receptors for the Fc domain of immunoglobulin G (IgG), so called Fc gamma receptors (FcγRs), link the specificity of IgG with potent effector functions of the innate immune system. FcγRs comprise a family of activating (FcγRI, FcγRIIa, and FcγRIIIa) and inhibiting (FcγRIIb) receptors that are differentially expressed on innate immune cells such as natural killer (NK) cells, monocytes, dendritic cells, neutrophils, and granulocytes (1–3). During an infection, these receptors play an important role in activating IgG-induced protective inflammatory processes and regulating immune responses (4–7).

Increasing evidence support an important role for FcγR-mediated effector functions, in particular antibody-dependent cellular cytotoxicity (ADCC), in HIV-1-specific immunity (6, 8). In adults, ADCC has been associated with a reduced risk of HIV-1 acquisition in the RV144 vaccine trial, whereas in infants born to HIV-1 infected mothers, passively acquired ADCC activity associated with reduced mortality (9). Similarly, ADCC responses associate with slower disease progression in HIV-1 infected adults (10–14).

In the periphery, CD56^dim^FcγRIIIa^bright^ NK cells are the primary effectors of ADCC responses (15). During a chronic HIV-1 infection, however, shedding of FcγRIIIa from the surface of cytotoxic CD56^dim^FcγRIIIa^bright^ NK cells together with an increase in the CD56^neg^FcγRIIIa^bright^ NK cell subset leads to a significant reduction in NK cell function (16, 17). It is unknown whether this loss in NK cell-mediated ADCC capacity is compensated for by other cytotoxic cells, where FcγR expression is induced upon immune cell activation. For chronic hepatitis C virus or Epstein Barr virus infections, for instance, it has been shown that FcγRIIIa expression is induced on an effector memory CD8^+^ T cell subset (18, 19). This FcγRIIIa^+^CD8^+^ T cell subset acquires NK cell-like functional properties, including ADCC activity accompanied by the release of pore forming perforin and serine protease granzyme B (18, 19). The presence and role of this CD8^+^ T cell subset in HIV-1 infection is currently undefined.

In addition to the effect of an actively replicating virus on FcγRIIIa expression, host genetics also contribute to variability of FcγRIIIa expression and/or ADCC responses. *FCGR3A* copy number variation directly correlates with the surface density of FcγRIIIa, with individuals bearing a single *FCGR3A* copy and correspondingly lower FcγRIIIa surface densities, having reduced ADCC responses compared to individuals with two or more gene copies (20). In addition, a phenylalanine (F) to valine (V) substitution at amino acid 158 in the proximal Ig-like domain of FcγRIIIa confers increased binding for IgG1, IgG3, and IgG4, which has been associated with higher NK cell activation and ADCC responses (21–23). Unlike *FCGR3A* copy number and the FcγRIIIa-F158V variant, a deletion of a copy number variable region (CNR) encompassing *FCGR3B* and *FCGR2C*—known as CNR1—does not affect FcγRIIIa directly (24). However, it juxtaposes the 5′-regulatory sequences of *FCGR2C* with the open reading frame of *FCGR2B*, creating a chimeric *FCGR2B'* gene (25). This results in the expression of the inhibitory FcγRIIb on NK cells where it regulates FcγRIIIa-mediated ADCC responses (25, 26).

*FCGR* variants are rarely adjusted for in studies that compare NK cell-mediated ADCC capacity between HIV-positive and HIV-negative individuals. Moreover, it is unclear if the altered immune milieu accompanying an HIV-1 infection modulates the functional consequences of the aforementioned variants. In this study, we sought to characterize FcγRIIIa expression on cytotoxic lymphocytes—NK cells and CD8^+^ T cells—and associated ADCC responses in healthy donors and viraemic HIV-1 individuals matched for *FCGR* genetic variants.

## Materials and Methods

### Cohort

All study participants were black South Africans recruited from the city of Johannesburg, Gauteng province, South Africa (Table 1). Self-reported HIV-1 uninfected individuals who did not have an acute or chronic illness at the time of sample collection were prospectively recruited from the National Institute for Communicable Diseases as healthy controls. Viraemic, treatment naïve HIV-1 infected individuals were identified from an existing cohort recruited from hospitals in Johannesburg and Soweto. This study was carried out in accordance with the recommendations of the National Health Research Ethics Council (NHREC) of the South African Department of Health. The protocol was approved by the University of the Witwatersrand Ethics Committee (Ethics clearance certificate no. M1511102). All participants provided written informed consent in accordance with the Declaration of Helsinki.

**Table 1:** Clinical and demographic characteristics of study cohort.

	**HIV-1 negative**	**HIV-1 positive**	***P*-value**
***N***	23	23	
Age [mean (SD)]	36.3 [8.5]	40.2 (8.8)	0.143^*^
Gender [% Females]	69.6	82.6	0.491^*^
**HIV-1 VIRAL LOAD [MEDIAN (IQR)]**			
158FF	–	6,931 (3,598–15,090)	0.563^†^
158FV/VV	–	4,732 (1,134–11,430)	
**CD4 T CELL COUNT [MEAN (*****SD*****)]**			
158FF	–	508 (267)	0.629^†^
158FV/VV	–	562 (261)	
**OTHER DISEASES OR INFECTIONS**			
*Mycobacterium tuberculosis*	0 (0%)	2 (8.7%)	

* *Comparison between HIV-1 negative and HIV-1 positive groups*.

† *Comparison between genotypes*.

### *FCGR* Variant Genotyping

Genomic DNA was isolated from ethylenediaminetetraacetic acid (EDTA)-anticoagulated whole blood. Study participants were genotyped for *FCGR* variants using the *FCGR*-specific multiplex ligation-dependent probe amplification (MLPA) assay (MRC Holland, Amsterdam, The Netherlands) as previously described (27, 28). This assay detects the genomic copy number of *FCGR2A, FCGR2B, FCGR2C, FCGR3A*, and *FCGR3B* and functional allelic variants that include FcγRIIa-H131R (alias H166R, c.497A>G, rs1801274); FcγRIIb-I232T (c.695T>C, rs1050501), FcγRIIIa-F158V (alias F176V, c.634T>G, rs396991), FcγRIIIb-HNA1a/b/c, *FCGR2C* gene expression variants c.169T>C (X57Q) and c.798+1A>G (rs76277413), and the *FCGR2B/C* promoter variants c.-386G>C (rs3219018) and c.-120T>A (rs34701572) in two multiplex reactions. Capillary electrophoresis was used to separate the MLPA assay amplicons on an ABI Genetic Analyzer 3500. Data were analyzed with Coffalyser.NET software created by the MLPA assay manufacturer, MRC Holland.

### Monoclonal Antibodies and Flow Cytometric Analysis

The following monoclonal antibodies were used: CD3-PerCP (SK7), CD56-AF647 (B159), CD8-APC-H7 (SK1), CD16-PE (3G8), CD16-FITC (NKP15), CD32-FITC (2B6), and CD32-PE (FLI8.26). All antibodies were obtained from BD Biosciences (San Jose, CA). Dead cells were labeled with the BD Horizon™ Fixable Viability Stain 510. Samples were acquired on a BD Fortessa X20 (BD biosciences) and analyzed on FlowJo version 9.8.1 software (Tree Star, San Carlos, CA). Fluorescence-minus-one (FMO) controls were used to set the appropriate gates for analyses.

### Isolation of Specific Cell Populations

Peripheral blood mononuclear cells (PBMCs) were isolated from EDTA-anticoagulated whole blood using Ficoll-Paque™ PLUS density gradient centrifugation (GE Healthcare) and stored at −80°C. NK cells and CD8^+^ T cells were positively selected from overnight rested PBMCs using MACS® magnetic cell separation technology (Miltenyl Biotec). To limit the presence of NK cells in the enriched CD8^+^ T cell preparation, NK cells were first isolated from PBMCs using CD56^+^ beads prior to isolation of CD8^+^ T cells with CD8^+^ beads. Viability and number of each participant's NK and CD8^+^ T cells were determined by trypan blue exclusion through direct cell counting on a haemocytometer. The purity of enriched cell fractions was determined by flow cytometric phenotyping assays.

### Antibodies

ADCC capacity was evaluated using pooled IgG isolated from the HIV-1 infected individuals included in the study. IgG was isolated from plasma using the Melon Gel IgG Purification kit according to the manufacturer's instructions (Thermo Scientific) and quantitated using the Bicinchoninic acid (BCA) assay. HIV immune globulin (HIVIG; NIH AIDS reagent program) was used as a positive control.

### ADCC

NK cells and CD8^+^ T cells both utilize the pore forming perforin and serine protease granzyme B during cytotoxic responses. It is therefore possible to evaluate ADCC responses of both NK cells and CD8^+^ T cells with the HIV-specific GranToxiLux assay (15). In brief, CEM.NK^R^.CCR5 cells were coated with recombinant HIV-1 ConC gp120, followed by opsonisation with HIV-1-specific antibodies and incubation for 45 min in the presence of NK cells or CD8^+^ T cells at an effector-to-target (E:T) ratio of 10:1. The optimal concentration of isolated IgG (30 μg/ml) was determined empirically. Samples were acquired on BD Fortessa X20 flow cytometer and data analyzed on FlowJo version 9.8.1 software (Tree Star, San Carlos, CA). Granzyme B activity in the absence of any antibody (background killing) was determined for each subject—NK cells and CD8^+^ T cells—and subtracted from granzyme B activity in the presence of antibody. The inter assay coefficient of variation, as calculated from a HIVIG-specific ADCC response measured for a single donor included in every run, was <10%.

**Figure 1:**
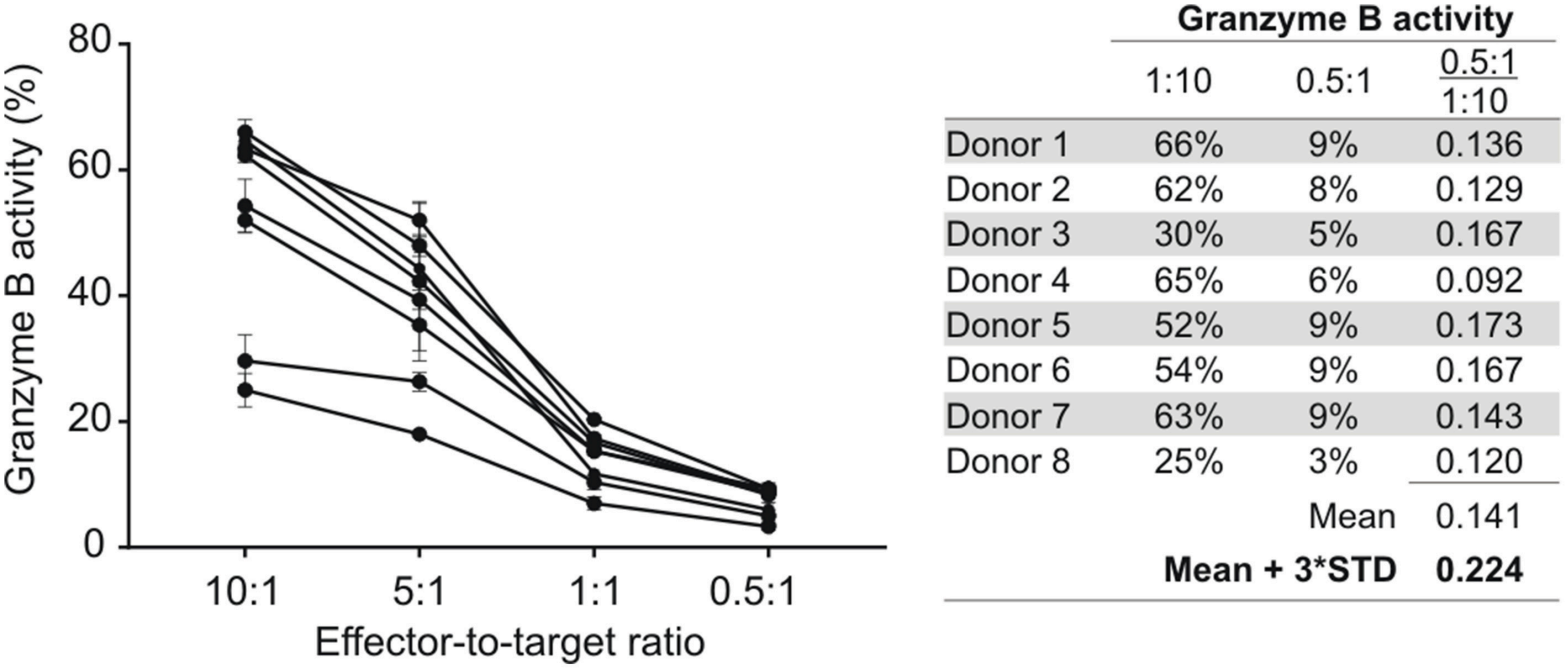
NK cell-mediated ADCC responses at different effector-to-target (E:T) cell ratios. NK cells isolated from eight HIV-1 negative donors were tested at different E:T ratios in an HIV-specific GranToxiLux assay using an ADCC-mediating monoclonal antibody (A32) at 2.5 μg/ml. The mean fraction of Granzyme B activity observed at an E:T of 0.5:1 relative to 10:1 was used to calculate a 99% confidence level cut-off value for NK cell-mediated ADCC responses at an E:T of 0.5:1. Data points represent the mean of triplicate measures and the standard deviation indicated by error bars.

To assess CD8^+^ T cell mediated granzyme B responses, HIV-1 positive donors with <5% NK cell contamination in their enriched CD8^+^ T cell fractions were identified and selected for further analysis. This proportion of NK cells equates to a <0.5:1 NK-to-target ratio in a 10:1 CD8-to-target preparation. In our experience, the NK cell-mediated granzyme B activity at 0.5:1 is on average 14.1% (standard deviation [SD]: 2.8%) of that observed for NK cells at a ratio of 10:1 (Figure 1). Using these data, a positivity threshold for CD8^+^ T cell-mediated granzyme B responses was calculated relative to autologous NK cell-mediated granzyme B responses whereby the mean granzyme B activity for autologous NK cells (E:T = 10:1) was multiplied with 22.4% (14.1% + 3 × SD:2.8%).

### Statistics

All statistical analyses were performed using GraphPad Prism 7 software version 7.04 (GraphPad Software). *P*-values from 2-tailed tests <0.05 were considered statistically significant. The significance of differences between unpaired data sets were analyzed with the Mann-Whitney U tests and paired data sets with the Wilcoxon matched-pairs signed rank test. The significance of differences between more than two data sets were analyzed using Kruskal-Wallis tests. Correlation analyses of data between two groups were assessed using the non-parametric Spearman rank correlation coefficient. To determine the role of FcγRIIIa-F158V alleles in ADCC responses, the V allele was studied under a dominant model due to the low prevalence of VV homozygotes (29).

## Results

### Study Population

**Figure 2:**
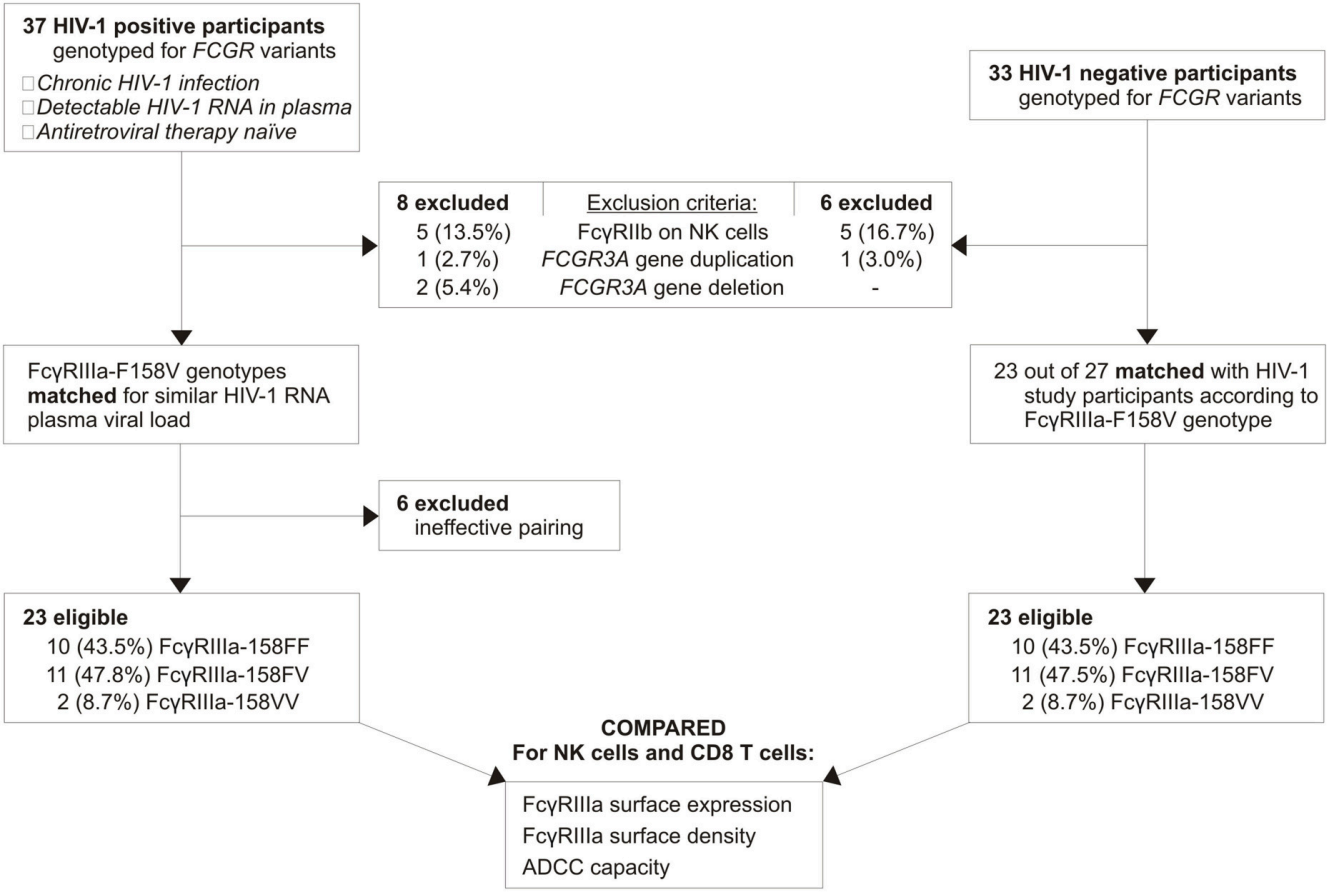
Study participant selection.

Thirty-seven chronic HIV-1 infected, viraemic, and antiretroviral naïve individuals with sufficient sample available were identified from an existing cohort of HIV-1 positive individuals (Figure 2). Following *FCGR* genotyping, seven individuals were excluded due to the possession of an *FCGR3A* gene duplication (*n* = 1), *FCGR3A* gene deletion (*n* = 2), or CNR1 deletion that results in the expression of the inhibitory FcγRIIb on NK cells (*n* = 4). To ensure that individuals carrying an undetectable CNR1 deletion—possess a duplication of this region on a single chromosome and a deletion on the other—were also excluded, expression of FcγRIIb on NK cells was monitored using flow cytometry. During the eligibility screening of individuals, such an individual was indeed identified and excluded from the study; thus, five HIV-1 positive individuals in total expressed FcγRIIb on their NK cells and were excluded.

Within the HIV-1 positive group, FcγRIIIa-158FF donors were paired with FcγRIIIa-158FV donors according to their HIV-1 RNA plasma viral load. Twenty-three HIV-1 positive individuals were eligible for further analysis, with the FcγRIIIa-F158V genotype distribution closely resembling that observed in the general black population from the same region in South Africa (29). As a comparative group, thirty-three HIV-1 negative individuals were genotyped for *FCGR* variants. Six individuals were excluded due to the possession of an *FCGR3A* duplication (*n* = 1) or CNR1 deletion (*n* = 5). Twenty-three eligible HIV-1 negative individuals were subsequently paired with HIV-1 positive individuals based on the FcγRIIIa-F158V genotype. None of the study participants expressed the activating FcγRIIc on their NK cells, as determined by the *FCGR2C* c.798+1A>G splice-site variant, or carried the *FCGR3A* intragenic haplotype previously associated with increased FcγRIIIa surface density (26, 30).

Age and gender did not differ significantly between HIV-1 negative and positive donors (Table 1). Two HIV-1 positive individuals had tuberculosis, of which one was an FcγRIIIa-158FF donor and the other an FcγRIIIa-158FV donor. No other infections were noted for these patients. Human cytomegalovirus (HCMV) infection status was not determined for study participants. However, the prevalence is likely 100% for the HIV-1 positive individuals and >85% for HIV-1 negative individuals as observed in other cohorts in rural and urban South Africa [(31) and Tiemessen, unpublished data]. Other sexually transmitted infections were not tested for in the study cohort.

### FcγRIIIa Expression on NK Cell Subsets

**Figure 3:**
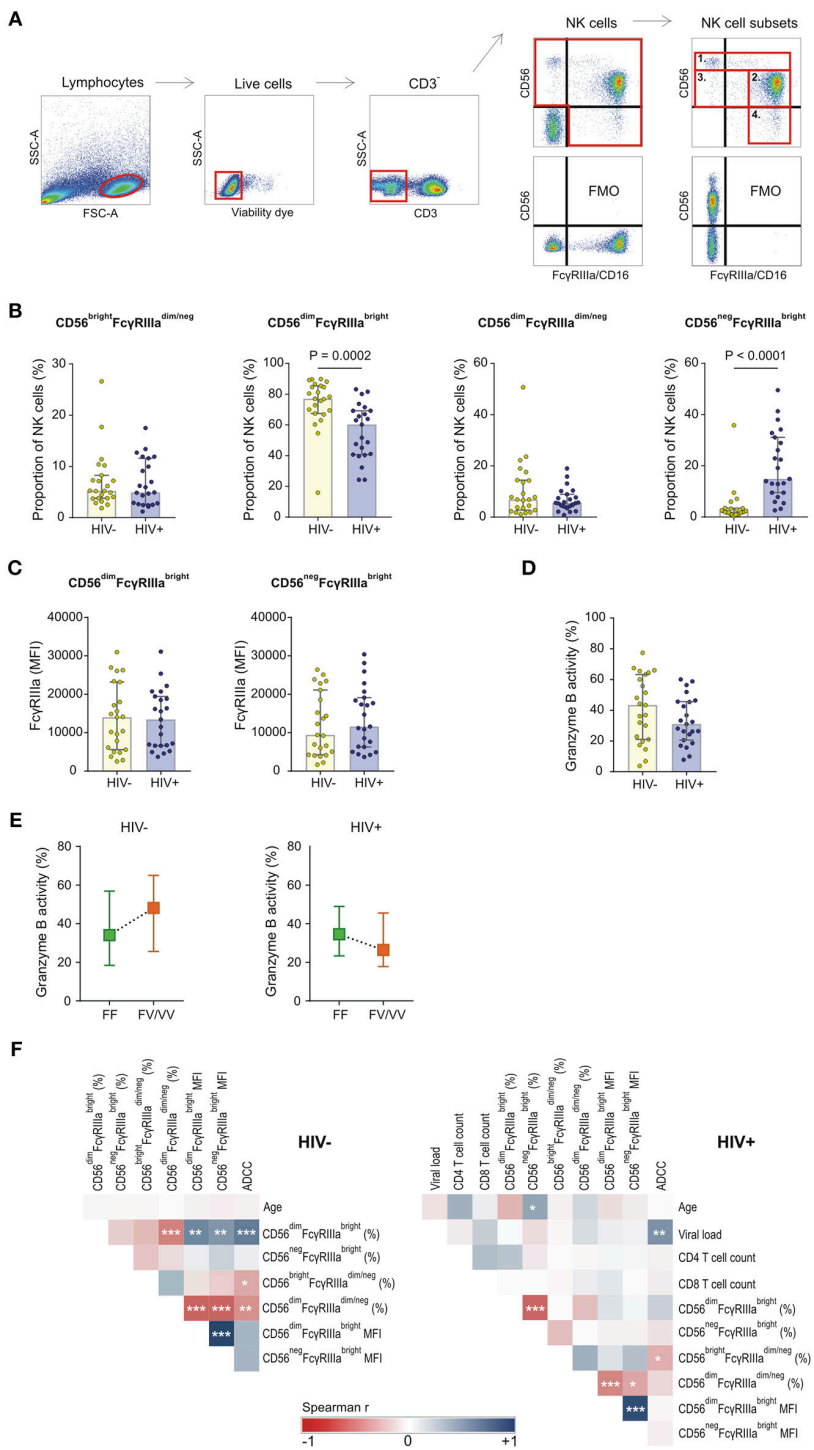
FcγRIIIa expression on NK cell subsets in HIV-1 uninfected and HIV-1 infected individuals matched for *FCGR* genetic variants. **(A)** Gating strategy for defining four NK cell subsets: 1. CD56^bright^FcγRIIIa^dim/neg^, 2. CD56^dim^FcγRIIIa^bright^, 3. CD56^dim^FcγRIIIa^dim/neg^, and 4. CD56^neg^FcγRIIIa^bright^; **(B)** Comparison of NK cell subsets between HIV-1 uninfected and infected individuals; **(C)** FcγRIIIa surface density on FcγRIIIa^bright^ NK cell subsets; **(D)** ADCC activity of NK cells at a target-to-effector cell ratio of 10:1 with isolated IgG pooled from HIV-1 study participants; **(E)** Median ADCC responses for individuals homozygous for the FcγRIIIa-158F allele and individuals bearing at least one FcγRIIIa-158V allele; **(F)** Correlation analysis between demographic, clinical, phenotypic and functional variables in HIV-1 uninfected and HIV-1 infected individuals (^***^P < 0.001; ^**^P < 0.01; ^*^P < 0.05).

NK cell subsets were defined based on the relative surface expression of CD56 and FcγRIIIa. Four subsets were identified that include CD56^bright^FcγRIIIa^dim/neg^, CD56^dim^FcγRIIIa^bright^, CD56^neg^FcγRIIIa^bright^, and CD56^dim^FcγRIIIa^dim/neg^ (Figure 3A). The distribution of these subsets within the NK cell population differed significantly between HIV-1 positive and HIV-1 negative donors. Compared to the HIV-1 negative group, the HIV-1 positive group had a smaller median proportion of CD56^dim^FcγRIIIa^bright^ cells (60.2 vs. 77.0%, *P* = 0.0002, Figure 3B) that was offset by a larger median proportion of CD56^neg^FcγRIIIa^bright^ cells (14.9 vs. 2.1%, *P* < 0.0001, Figure 3B). The inverse relationship between the NK cell subsets in HIV-1 positive individuals, but not HIV-1 negative individuals, was further observed in a correlation analysis (*R* = −0.633, *P* = 0.001, Figure 3F). The relative proportions of CD56^bright^FcγRIIIa^dim/neg^ and CD56^dim^FcγRIIIa^dim/neg^ did not differ significantly between HIV-1 positive and HIV-1 negative donors. In contrast to the observed differences in NK cell subsets, FcγRIIIa surface density on neither cytotoxic CD56^dim^FcγRIIIa^bright^ nor CD56^neg^FcγRIIIa^bright^ NK cells differed between the two groups (*P* = 0.948 and *P* = 0.486, respectively; Figure 3C).

### NK Cell-Mediated ADCC Responses

The capacity of CD56^+^ NK cells to mediate ADCC was tested in an HIV-1-specific granzyme B assay in the presence of pooled IgG isolated from HIV-1-infected South Africans. ADCC responses, measured as granzyme B activity in target cells, were reduced in the HIV-1 positive group compared to the HIV-1 negative group, although not statistically significantly different (31.0 vs. 43.3%, *P* = 0.184, Figure 3D). In both groups, the ADCC responses were affected by the FcγRIIIa-F158V variant (Figure 3E). Healthy donors bearing at least one V allele had a higher median NK cell-mediated granzyme B activity compared to those homozygous for the F allele (48.1 vs. 34.1%, *P* = 0.284). This trend was, however, not observed in the HIV-1 positive group. In contrast, HIV-1 positive donors bearing at least one V allele had reduced granzyme B activity compared to those homozygous for the F allele (26.4 vs. 34.6%, *P* = 0.522).

ADCC responses of both HIV-1 negative and HIV-1 positive donors negatively correlated with the proportion of immunoregulatory CD56^bright^FcγRIIIa^dim/neg^ cells (*R* = −0.486, *P* = 0.019; and *R* = −0.454, P = 0.030, respectively; Figure 3F). Furthermore, ADCC responses of HIV-1 negative donors, but not HIV-1 positive donors, positively correlated with the proportion of CD56^dim^FcγRIIIa^bright^ cells (*R* = 0.665, *P* = 0.0005; and *R* = 0.233, *P* = 0.284, respectively; Figure 3F). FcγRIIIa expression levels on the cytotoxic CD56^dim^FcγRIIIa^bright^ cell subset did not correlate with ADCC responses in either HIV-1 negative or HIV-1 positive group.

### Expression of FcγRIIIa on CD8^+^ T Cells

**Figure 4:**
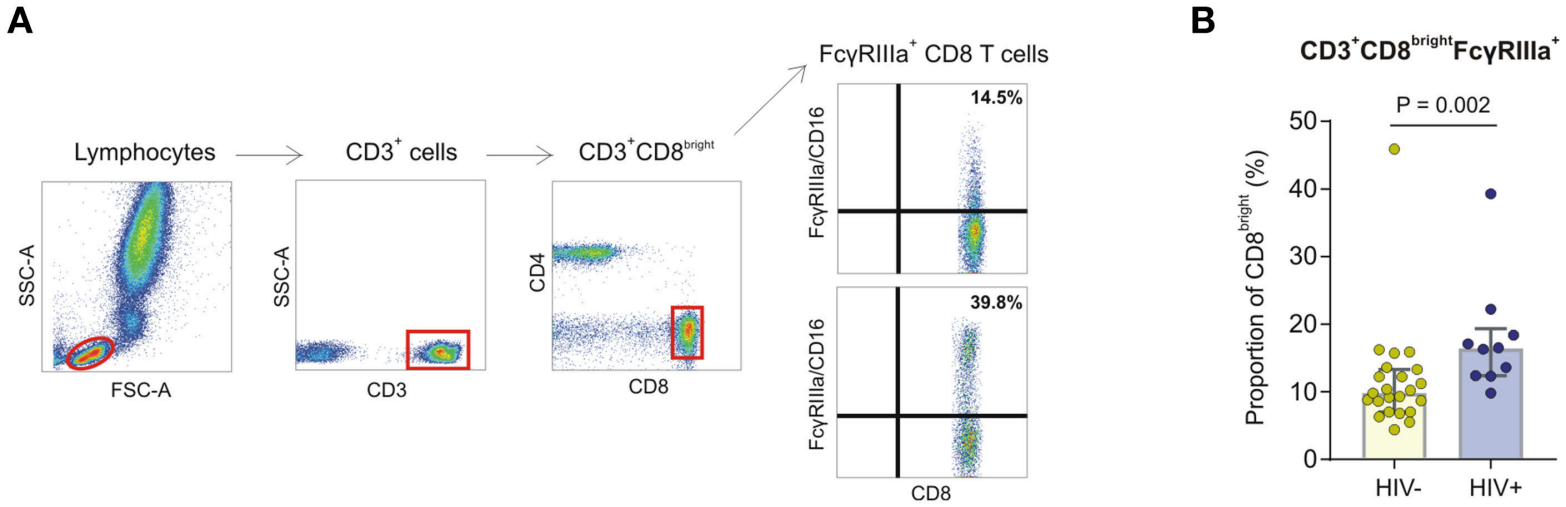
FcγRIIIa expression on CD8 T cells in whole blood obtained from HIV-1 negative and HIV-1 positive donors. **(A)** Gating strategy showing two representative individuals with low and high proportions of FcγRIIIa^+^CD8^bright^ T cell subsets. **(B)** Frequencies of FcγRIIIa^+^CD8^bright^ T cells in whole blood isolated from HIV-negative healthy controls (*n* = 23) and HIV-infected individuals (*n* = 10).

During the course of characterizing FcγRIIIa expression on peripheral leukocytes in whole blood obtained from a preliminary cohort of HIV-1 negative and HIV-1 positive donors, we observed a CD8^bright^ T cell subset expressing FcγRIIIa in both groups (Figure 4). The proportion of FcγRIIIa^+^CD8^bright^ T cells within the CD8^bright^ T cell population varied extensively, ranging from 4.4 to 45.9%, with the median proportion significantly higher in the HIV-1 infected group compared to the healthy control group (17.8 vs. 9.8%, *P* = 0.002; Figure 4).

**Figure 5:**
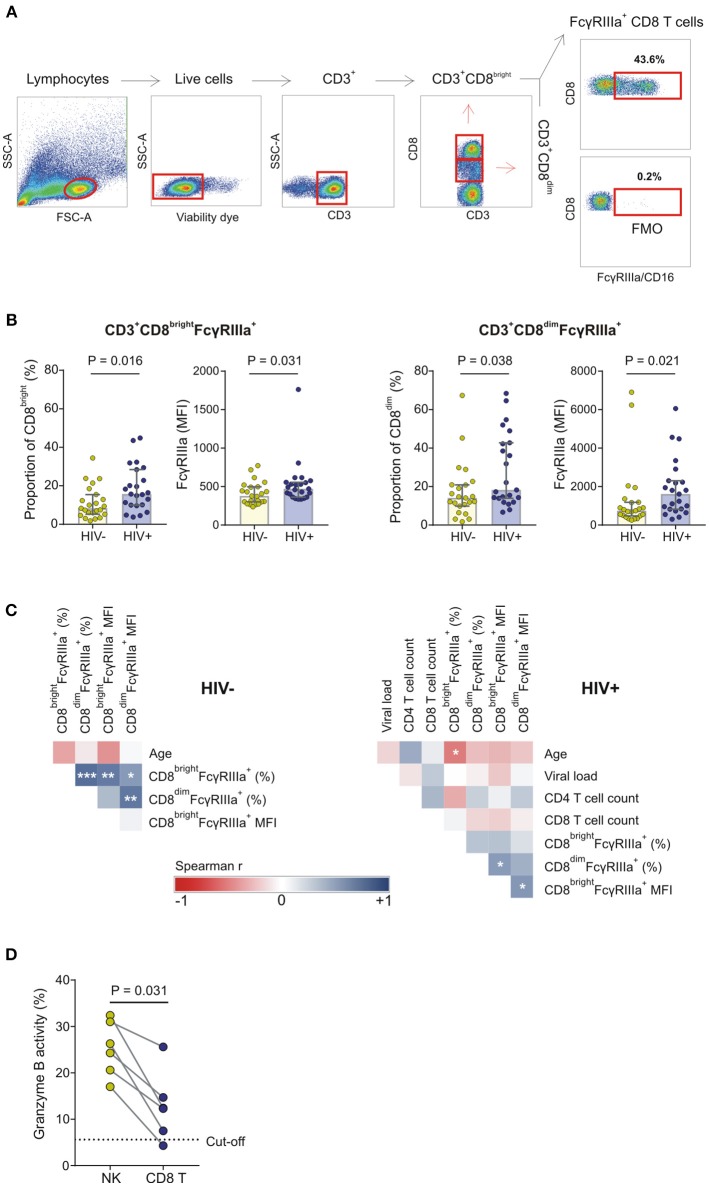
FcγRIIIa expression on CD8 T cells in HIV-1 uninfected and HIV-1 infected individuals matched for *FCGR* genetic variants. **(A)** Gating strategy; **(B)** The proportion of FcγRIIIa^+^CD8^bright^ and FcγRIIIa^+^CD8^dim^ T cells and corresponding median fluorescence intensity (MFI) of FcγRIIIa on these FcγRIIIa^+^CD8^+^ T cell subsets in a cohort of HIV-1 uninfected and infected individuals; **(C)** Correlation analysis between demographic, clinical, phenotypic, and functional variables in HIV-1 uninfected and HIV-1 infected individuals; **(D)** CD8^+^ T cell-mediated ADCC responses of HIV-1 positive donors relative to autologous NK cell-mediated ADCC responses at the same effector-to-target cell ratio (^***^P < 0.001; ^**^P < 0.01; ^*^P < 0.05).

In the present validation study, two CD8^+^ T cell subsets are identified, designated CD8^bright^ and CD8^dim^ (also referred to as CD8^high^ and CD8^low^), according to the relative expression of CD8 on CD3^+^ T cells (Figure 5). The CD8^dim^ subset accounted for 13.9 and 13.4% of the total CD8^+^ T cell population in HIV-1 negative and HIV-1 positive donors, respectively (*P* = 0.978). In agreement with our observations in whole blood, 2–44.8% of CD8^bright^ cells and 2–68.4% of CD8^dim^ cells expressed FcγRIIIa. Compared to its FcγRIIIa^+^CD8^bright^ counterpart, the FcγRIIIa^+^CD8^dim^ subset expressed significantly higher levels of FcγRIIIa in both the HIV-1 positive and negative group (471 vs. 1610, *P* < 0.001; and 377 vs. 740, *P* < 0.001, respectively; data not shown).

Compared to the HIV-1 negative group, the HIV-1 positive group had a significantly higher median proportion of FcγRIIIa^+^CD8^bright^ and FcγRIIIa^+^CD8^dim^ T cell subsets (15.7 vs. 8.3%; *P* = 0.016 and 18.2 vs. 14.1%; *P* = 0.038, respectively) and correspondingly higher median FcγRIIIa surface densities on both FcγRIIIa^+^CD8^+^ T cell subsets (471 vs. 377, *P* = 0.031; and 1,610 vs. 740, *P* = 0.021, respectively) (Figure 5B). The proportion of FcγRIIIa^+^CD8^bright^ and FcγRIIIa^+^CD8^dim^ cells positively correlated in the HIV-1 negative group (*R* = 0.671, *P* = 0.001; Figure 5C). However, this relationship was not observed for the HIV-1 positive group. In the latter group, neither the presence nor levels of FcγRIIIa correlated with CD4^+^ T cell count or HIV-1 plasma viral load (Figure 5C). However, the proportion of FcγRIIIa on CD8^bright^ T cells negatively correlated with age in both groups, although this correlation was only statistically significant for the HIV-1 positive group (*R* = −0.510, *P* = 0.013; Figure 5C).

### CD8^+^ T Cells Mediate ADCC

To assess CD8^+^ T cell-mediated ADCC responses, CD8^+^ T cells were positively selected from PBMCs after depletion of NK cells. The proportion of potential contaminating NK cells was determined using flow cytometry and six HIV-1 positive donors were identified with <5% NK cells in their enriched CD8^+^ T cell fractions (mean: 1.3%; range: 0.3–2.9%). A threshold for positive CD8^+^ T cell-mediated granzyme B responses (5.6%) was calculated based on the corresponding NK cell granzyme B responses at an NK-to-target ratio of 10:1 (described in section Materials and Methods). Five out of six donors had positive CD8^+^ T cell-mediated granzyme B responses that ranged from 7.5 to 25.6% (mean: 14.5%; Figure 5E). Overall, the CD8^+^ T cell-mediated granzyme B activity was comparatively lower than that observed for their NK cell counterparts (*P* = 0.031).

## Discussion

This study set out to comprehensively characterize FcγRIIIa variability and its effect on ADCC responses in HIV-1 positive and HIV-1 negative South African individuals, the population that bears the largest HIV-1 epidemic. Accurate assessment of FcγRIIIa variability requires careful selection of study participants to exclude confounding genetic variables that modify FcγRIIIa expression and FcγRIIIa-mediated cell activation. Study participants within the comparative groups were further matched for the FcγRIIIa-F158V variant that not only alters ADCC responses, but also affects measurements of FcγRIIIa surface expression with the most commonly used anti-FcγRIIIa antibody, clone 3G8 (32).

In comparing a cohort of Black South African HIV-1 negative and HIV-1 positive donors matched for genotypic variants, we confirmed previously described significant differences in the distribution of NK cell subsets, while differences in FcγRIIIa surface density on cytotoxic NK cells were not observed. NK cell-mediated ADCC responses of HIV-1 positive donors were both reduced and differentially affected by the FcγRIIIa-F158V variant compared to HIV-1 negative donors. In addition, FcγRIIIa expression was identified on a subset of cytotoxic CD8 T cells where it potentially contributes to HIV-1-specific ADCC responses in a granzyme B-dependent manner.

In healthy individuals, NK cells typically comprise a dominant CD56^dim^FcγRIIIa^bright^ population and minor populations that include CD56^bright^FcγRIIIa^dim/neg^, CD56^dim^FcγRIII^dim/neg^ and CD56^neg^FcγRIIIa^bright^. Perturbation of NK cell subsets in the presence of an HIV-1 infection has been extensively described (16, 17, 33). In HIV-1 infected individuals, the cytotoxic CD56^dim^FcγRIIIa^bright^ subset contracts with an associated expansion of the CD56^neg^FcγRIIIa^bright^ subset. The latter is characterized by higher levels of inhibitory NK cell receptors, lower levels of natural cytotoxicity receptors, and reduced secretion of cytokines compared to the CD56^dim^FcγRIIIa^bright^ subset (16, 17). This hyporesponsive NK cell subset was similarly increased in the South African HIV-1 positive cohort; however, it did not associate with HIV-1 viral load as observed by Mavilio et al. (16, 34). The lack of an association with HIV-1 viral load may be explained by an ~5- to 6-fold lower median HIV-1 plasma viral load of the South African cohort compared to the other cohorts. Moreover, in the present study, NK cell subsets were studied for overnight rested PBMCs as opposed to freshly isolated negatively-selected NK cells.

The dysregulation of NK cell subsets is typically associated with reduced ADCC responses in HIV-1 infected individuals (16, 17, 35, 36). In the present study, lower ADCC responses were similarly observed for HIV-1 infected individuals. Since ADCC capacity was studied for CD56^+^ NK cells it precluded an analysis of the association between the CD56^neg^FcγRIIIa^bright^ subset and ADCC responses, but not for the CD56^dim^FcγRIIIa^bright^ and CD56^bright^FcγRIIIa^dim/neg^ subsets. A negative correlation observed between the CD56^bright^FcγRIIIa^dim/neg^ subset and ADCC responses for both HIV-1 negative and positive donors would suggest that ADCC capacity is similarly affected by the immunoregulatory CD56^bright^FcγRIIIa^dim/neg^ subset in both groups. However, as demonstrated by a positive correlation between the cytotoxic CD56^dim^FcγRIIIa^bright^ subset and ADCC responses for HIV-1 negative donors, but not HIV-1 positive donors, not all factors modulating ADCC capacity may be shared between healthy individuals and HIV-1 infected individuals.

Given the association of ADCC-mediating antibody responses with HIV-1 protective immunity, it could be hypothesized that genetic determinants of NK cell-mediated ADCC capacity, in particular the FcγRIIIa-F158V variant, may associate with HIV-1 acquisition risk or disease progression. The FcγRIIIa-158V isoform has greater avidity for complexes comprising IgG1, IgG3, and IgG4 than the FcγRIIIa-158F isoform and confers increased NK cell activation and ADCC responses in healthy individuals (21–23). Despite its potential contribution to ADCC responses, the FcγRIIIa-158V isoform is yet to be positively associated with HIV-1 acquisition and disease progression. On the contrary, the FcγRIIIa-158V isoform has been associated with an increased risk of HIV-1 infection (37), disease progression (37), and HIV-1-associated Kaposi's sarcoma (KS) and Cryptococcal disease (38, 39). Furthermore, homozygosity for the FcγRIIIa-158V allele associated with a higher rate of HIV-1 infection among vaccinated men in the VAX004 trial (40), while homozygosity for the FcγRIIIa-158F allele associated with greater protection from HIV-1 disease progression in male participants in the RV144 vaccine trial (41). Taken together, these findings are more indicative of FcγRIIIa-158V-mediated antibody-dependent enhancement of infection rather than improved ADCC responses to the benefit of the individual. Alternatively, it is possible that the functional consequence of the FcγRIIIa-F158V isoforms may be different in the presence of an HIV-1 infection and that a different mechanism(s) may underlie the aforementioned associations.

FcγRIIa/FcγRIIIb polymorphic variants, for example, show distinct differences in oxidative burst responses of resting neutrophils; however, once neutrophils were pre-activated with IFNγ and G-CSF these differences were no longer observed (42). Other non-FcγR genetic variants have also been shown to differentially affect gene expression or cytokine production of activated and resting immune cells (43). In the present study, we show a similar trend for the FcγRIIIa-F158V variant, higher ADCC responses for HIV-1 negative donors bearing the FcγRIIIa-158V allele compared to those homozygous for the FcγRIIIa-158F allele, whereas in HIV-1 positive donors this trend was lost or even slightly reversed. The effect of the variant on ADCC responses was, however, not significant in either group. It is possible that the independent effect size of the FcγRIIIa-F158V variant is too small to detect with the current sample size. Larger cohort studies that adjust for other NK cell activation and inhibitory receptors are required to further define the role of this variant in HIV-1 infection. Nonetheless, these findings may partially explain the inconclusive role of FcγRIIIa genetic variants in HIV-1-specific immunity [reviewed by Cocklin and Schmitz (44)].

In addition to modulating the function of an allelic variant, infection can lead to the induction of FcγRIIIa on other cytotoxic cells, including CD8^+^ T cells. An FcγRIIIa^+^CD8^+^ T cell population was first described in the 1980's and has since been characterized in the context of hepatitis C virus and Epstein Barr virus infections (18, 19, 45). These terminally differentiated CD8^+^ T cells belong to the T effector memory CD45RA^+^ lymphocyte subset, are perforin positive, directly mediate ADCC *ex vivo*, and increase *in vivo* during hyperlymphocytosis (18, 19). In addition to FcγRIIIa, this cell subset also has increased expression of other NK-like receptors including NKG2A, NKG2D, KIR2DL2/L3 and KIR2DL1/S1 when compared to FcγRIIIa^−^CD8^+^ T cells (46). The present study validates the expression of FcγRIIIa on cytotoxic CD8^+^ T lymphocytes and is in agreement with other studies that have consistently showed an increase in the proportion of FcγRIIIa^+^CD8^+^ T cells in the presence of a virus infection (18, 19, 46). Compared to CD8^bright^ cells, the surface density of FcγRIIIa was significantly higher on CD8^dim^ cells, a subset characterized by higher activation levels, increased cytotoxicity and increased cytokine production (47–49). The higher proportions of FcγRIIIa^+^CD8^+^ T cells in HIV-1-infected individuals suggests that HIV-1 in its own right is a driver of these cell expansions, whereas increasing age associated with reduced proportions of FcγRIIIa-expressing CD8^+^ T cells. The increase in FcγRIIIa expression on CD8^+^ T cells in HIV infection contrasts with decreased proportions of FcγRIIIa expressing NK cells. This suggests the development of an ADCC capacity by CD8^+^ T cells that could compensate to some extent for the reduction in NK cell ADCC function.

Attributing the otherwise innate cell function of ADCC to CD8^+^ T cells—mediated through expression of FcγRIIIa and engagement of HIV-1-specific antibodies—is reminiscent of other known examples of innate-like unconventional T cell populations. Among these are invariant NKT cells, mucosal-associated invariant T cells, and γδT cells, that recognize foreign/self-lipid presented by non-classical MHC molecules (50, 51). Another interesting unconventional CD8^+^ T cell subset is one with a prominent innate/memory phenotype identified by co-expression of eomesodermin (Eomes) and KIR/NKG2A (52). The current study highlights the addition of another unconventional CD8^+^ T cell population, capable of ADCC function, that warrants further investigation.

In conclusion, our findings underscore the importance of expanding studies of HIV-specific antibodies to include the influence of different host cell types that share expression of FcγRs (constitutive or induced), the respective functional cellular capabilities, as well as host genotypes, in the context of the presiding immune milieu which is altered as a consequence of chronic HIV infection. Continuing investigations are warranted to further define effector functions, cytokine production and activation status of these different FcγRIIIa^+^ NK and CD8 T cell subsets in similarly selected individuals.

## Author Contributions

NP recruited HIV-1 negative donors, performed the majority of the experiments, analyzed the data and wrote the manuscript. RL designed the study, contributed to the data analysis, and writing of the manuscript. BD contributed to the flow cytometry experiments. ZW and NM recruited HIV-1 positive donors, CT in her capacity as head of the laboratory, allocated funds toward the study, supervised the research, and provided the necessary infrastructure to perform the work.

### Conflict of Interest Statement

The authors declare that the research was conducted in the absence of any commercial or financial relationships that could be construed as a potential conflict of interest.

## Abbreviations

NK: natural killer cell
IgG: immunoglobulin G
HIV-1: human immunodeficiency virus 1
ADCC: antibody-dependent cellular cytotoxicity
*FCGR*: Fc gamma receptor gene
FcγR: Fc gamma receptor protein
CNR1: gene copy number variable region 1
EDTA: ethylenediaminetetraacetic acid
HNA: Human neutrophil antigen
MLPA: multiplex ligation-dependent probe amplification
FMO: flurescence minus one
PBMCs: peripheral blood mononuclear cells
KIR2DL2/L3: killer immunoglobulin-like receptor 2 DL2/L3
NKG2A/D: natural killer group 2A or−2D.
